# Role of an Iron-Dependent Transcriptional Regulator in the Pathogenesis and Host Response to Infection with *Streptococcus pneumoniae*

**DOI:** 10.1371/journal.pone.0055157

**Published:** 2013-02-20

**Authors:** Radha Gupta, Minny Bhatty, Edwin Swiatlo, Bindu Nanduri

**Affiliations:** 1 Department of Microbiology, University of Mississippi Medical Center, Jackson, Mississippi, United States of America; 2 Department of Basic Sciences, College of Veterinary Medicine, Mississippi State University, Mississippi State, Mississippi, United States of America; 3 Department of Medicine, University of Mississippi Medical Center, Jackson, Mississippi, United States of America; Charité-University Medicine Berlin, Germany

## Abstract

Iron is a critical cofactor for many enzymes and is known to regulate gene expression in many bacterial pathogens. *Streptococcus pneumoniae* normally inhabits the upper respiratory mucosa but can also invade and replicate in lungs and blood. These anatomic sites vary considerably in both the quantity and form of available iron. The genome of serotype 4 pneumococcal strain TIGR4 encodes a putative iron-dependent transcriptional regulator (IDTR). A mutant deleted at *idtr* (Δ*idtr*) exhibited growth kinetics similar to parent strain TIGR4 in vitro and in mouse blood for up to 48 hours following infection. However, Δ*idtr* was significantly attenuated in a murine model of sepsis. IDTR down-regulates the expression of ten characterized and putative virulence genes in nasopharyngeal colonization and pneumonia. The host cytokine response was significantly suppressed in sepsis with Δ*idtr*. Since an exaggerated inflammatory response is associated with a poor prognosis in sepsis, the decreased inflammatory response could explain the increased survival with Δ*idtr*. Our results suggest that IDTR, which is dispensable for pneumococcal growth in vitro, is associated with regulation of pneumococcal virulence in specific host environments. Additionally, IDTR ultimately modulates the host cytokine response and systemic inflammation that contributes to morbidity and mortality of invasive pneumococcal disease.

## Introduction

The Gram-positive bacterium *Streptococcus pneumoniae* (pneumococcus) is an opportunistic human pathogen whose primary niche is the human nasopharynx. In susceptible individuals pnuemococcus can invade other anatomic sites causing otitis media, pneumonia, bacteremia, and meningitis leading to significant morbidity and mortality [1]. The mechanisms of translocation of pneumococci from nasopharynx to sterile sites, and changes in its physiology to adapt to these different niches are still not clearly understood.

Several studies have shown that iron is an important nutrient required for pneumococcal growth and survival in vitro and in vivo [2]–[4]. Pneumococci can utilize various iron sources such as ferric and ferrous iron salts, hemoglobin, hemin, ferritin, and ferrioxamine [3]–[6]. The different anatomic sites of pneumococcal infection vary considerably in the quantity as well as the form of available iron sources. The nasopharynx is a markedly iron-restricted environment while blood has a comparatively high total iron level. Hemoglobin and ferritin are the main iron-containing molecules in the blood. Lactoferrin, transferrin, ferritin (released from cell turnover at mucosal surfaces) and possibly small amounts of hemoglobin and its breakdown products are potential iron sources in the respiratory tract. Xenosiderophores produced by nasopharyngeal commensals may be a source of iron for pneumococci during nasopharyngeal colonization [3]. Since pneumococci can replicate in different host environments with varying iron availability it is likely that pneumococci sense changes in iron availability in the host environment and regulate gene expression in response. We hypothesize that iron is potentially an important environmental signal which regulates expression of genes required for pneumococcal survival and virulence in the host.

Iron-dependent regulators (IdeRs) are metal-activated DNA-binding proteins found in a wide variety of bacteria. These proteins are transcriptional regulators which bind to specific DNA sequences in the promoter regions of genes that they regulate in an iron-dependent manner. The classical ferric-uptake regulator (Fur) of *Escherichia coli* is a well-characterized, iron-responsive regulator which represses transcription of multiple operons in response to intracellular levels of iron [7]. Homologs of Fur have been identified in several Gram-negative pathogens such as *Vibrio*, *Pseudomonas*, *Yersinia*, and *Neisseria* [8]–[12]. The functional homolog of Fur in Gram-positive pathogens is represented by a family of metal-responsive transcriptional regulators whose prototype is the diphtheria toxin repressor protein (DtxR). DtxR homologs have been identified in other bacteria such as *Streptomyces* spp., *Staphylococcus epidermidis*, *Mycobacterium smegmatis* and the spirochete *Treponema denticola* [13]–[16]. The genome of TIGR4, an invasive serotype 4 pneumococcal human isolate encodes a putative iron-dependent transcriptional regulator (IDTR) [17]. The present study was designed to evaluate the role of IDTR in the survival and pathogenesis of pneumococcus in different host environments. Since much of the pathology of pneumococcal infections is a consequence of host inflammatory responses we also examined the association between IDTR and host immune responses represented by a selected set of cytokines.

## Results

### Role of *idtr* in pneumococcal growth in vitro

**Figure 1:**
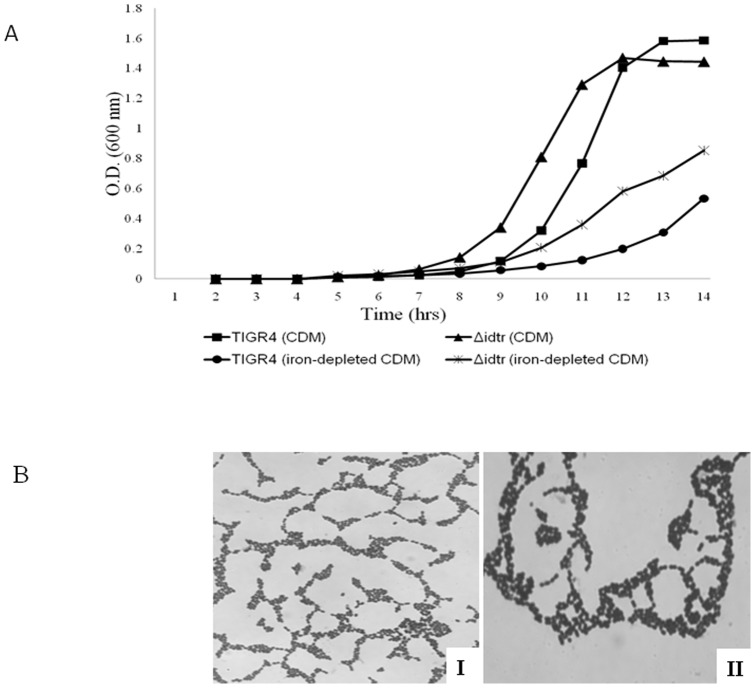
Growth of TIGR4 and Δ*idtr* and Gram stain morphology of Δ*idtr* in vitro. The growth of TIGR4 and Δ*idtr* in CDM and iron depleted CDM was monitored by measuring absorbance at 600 nm. B) The morphology of Δ*idtr* was observed in (I) Iron depleted CDM (II) CDM by Gram staining. The results shown are average of three independent experiments cells grown in iron.

The role of *idtr* in vitro in the presence or absence of free iron was examined. TIGR4 and Δ*idtr* exhibited similar growth kinetics in chemically-defined medium (CDM) and iron-depleted CDM. The deletion mutant had a shorter lag phase than TIGR4 but both attained similar cell density at stationary phase. Also, Δ*idtr* entered the exponential phase of growth slightly faster than TIGR4 in both CDM and iron-depleted CDM (Figure 1A). The microscopic appearance of Δ*idtr* cells was strikingly different from TIGR4. The mutant formed aggregates and clusters as contrasted with short chains and diplococci of the parent wild-type TIGR4 (Figure 1B).

### Role of *idtr* in growth and survival in a mouse model of sepsis

**Figure 2:**
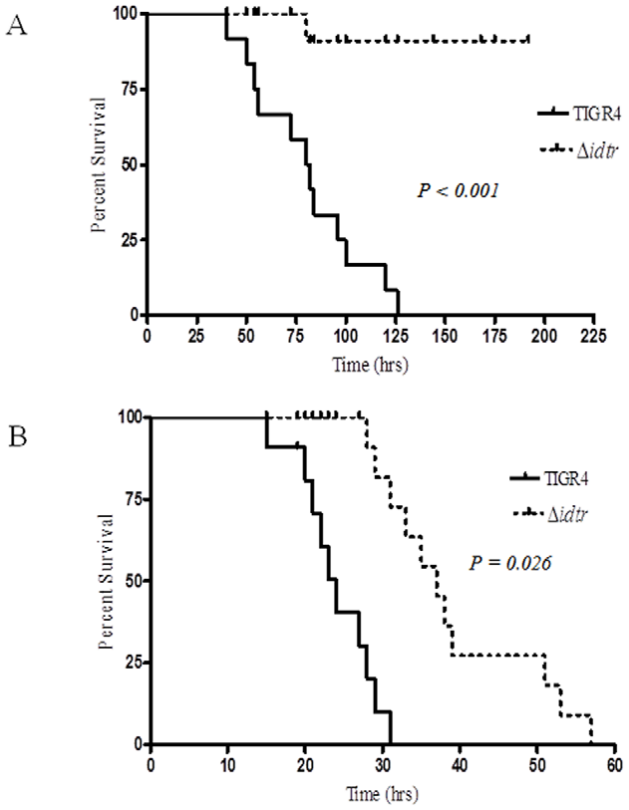
Survival of mice infected with TIGR4 and Δ*idtr*. CBA/CaHN-Btk*^xid^*/J mice were inoculated (A) intranasally with 10^6^ CFU and (B) intravenously with 10^5^ CFU of TIGR4 and Δ*idtr*. Kaplan Meier curves shown are a representative of triplicate experiments (n = 5 in each experiment).

**Figure 3:**
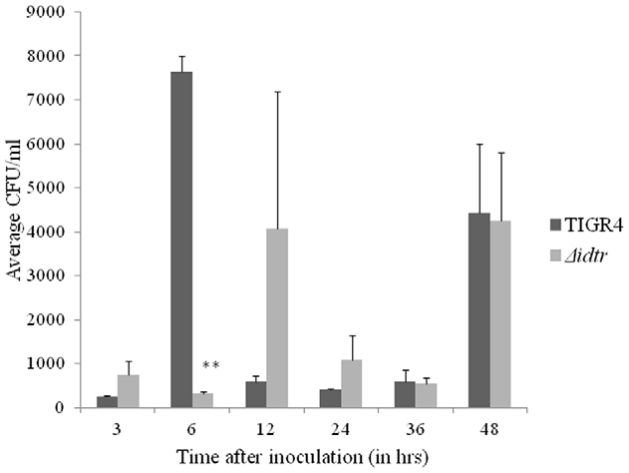
Average bacterial counts from mouse blood TIGR4 and Δ*idtr*. A group of 5 mice each were infected intravenously with 10^5^ CFU of TIGR4 or Δ*idtr*. Blood samples at different time points were plated to determine bacterial counts. The error bars represent standard error of mean. ^**^Significantly decreased as compared to TIGR4 infected blood counts (P<0.01).

The role of *idtr* in sepsis was evaluated using a mouse model. The Δ*idtr* mutant was significantly attenuated in a mouse model of sepsis induced by either intranasal or intravenous infection (Figure 2A and B). Although there was a significant survival advantage in mice infected i.v. with Δ*idtr* all mice eventually succumbed to infection. The kinetics of bacterial cell growth did not appear to vary greatly between TIGR4 and Δ*idtr* following i.v. infection (Figure 3). However, loss of *idtr* markedly attenuates the ability of pneumococcus to invade and cause fatal bacteremia from the nasopharyngeal epithelial surface.

### Expression of selected virulence genes in vitro and in vivo

**Figure 4:**
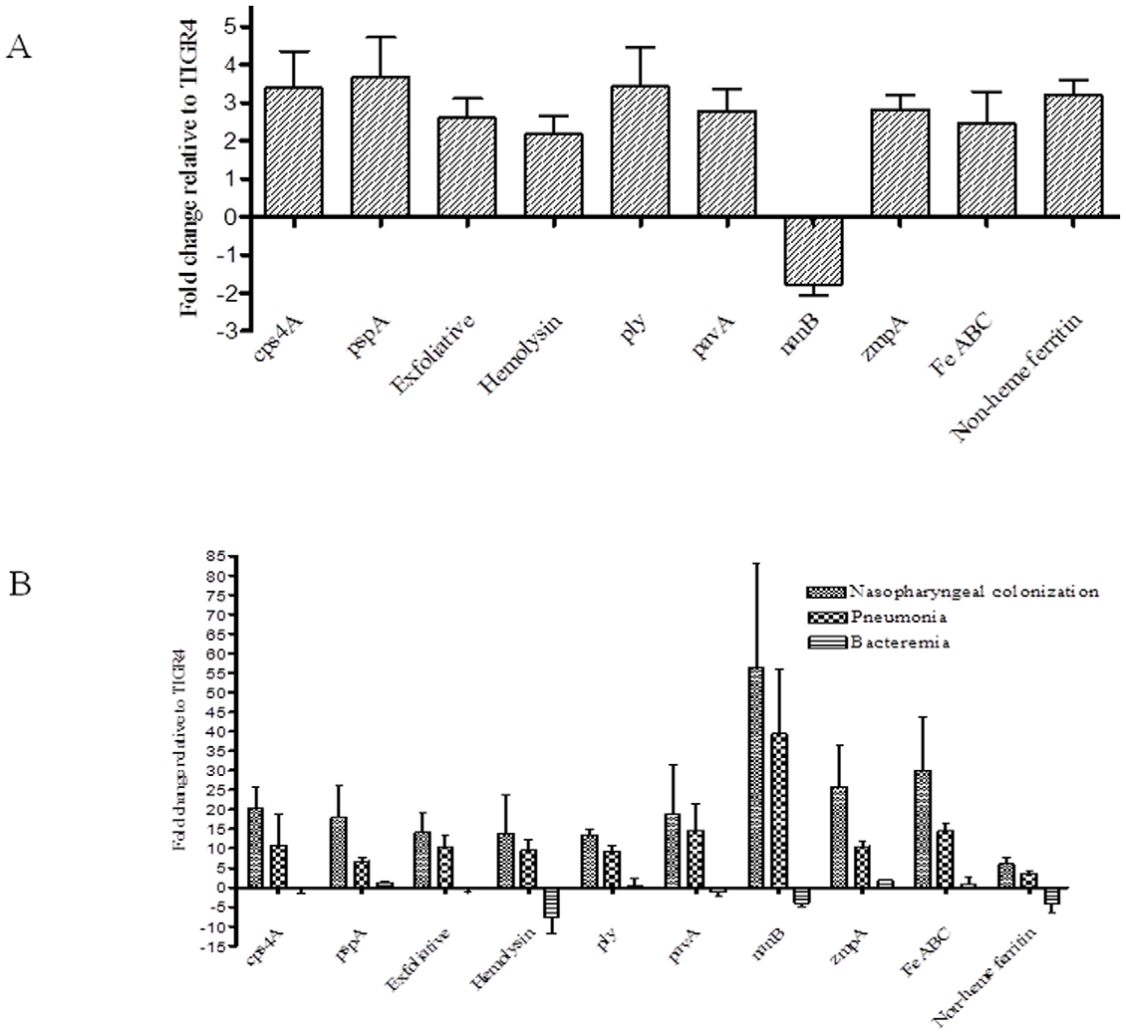
Pneumococcal gene expression in Δ*idtr* in vitro and in vivo. Expression of ten pneumococcal genes in Δ*idtr* relative to TIGR4 in CDM (A) and from nasopharyngeal washes, lung homogenates and blood samples (B) was quantified by RT-PCR. Each experiment was performed using three separate biological sample, each done in triplicate.

Expression of several well characterized and putative virulence genes in Δ*idtr* and TIGR4 in vitro and in vivo was evaluated. Transcripts of *cps4A*, *pspA*, *ply*, hemolysin, and non-heme ferritin were up-regulated in Δ*idtr* cells, while those of exfoliative toxin, iron ABC transporter and *pavA* remained essentially unchanged in vitro. Transcription of *nanB* was markedly repressed in the deletion mutant (Figure 4A).

The expression of these genes in vivo varied significantly at the three anatomical sites examined. During nasopharyngeal colonization, the transcription of all ten genes was up-regulated in the mutant. During pneumonia transcription of the genes in the mutant was less than that during colonization but still higher when compared to that in TIGR4. During bacteremia transcription was unchanged or slightly repressed in the mutant as compared to TIGR4 cells (Figure 4B).

### Affect of *idtr* on host cytokine response in intravenous sepsis model

Based on studies in humans and animal models, a panel of 14 cytokines (Table 1) were chosen [18]–[24] to evaluate the effect of *idtr* on the host innate immune response. At 48 hours after infection the concentration of all 14 cytokines that were tested was significantly decreased in the plasma samples of mice infected with Δ*idtr* as compared to those challenged intravenously with TIGR4 (Table 1).

**Table 1:** Average plasma cytokine/chemokine concentration in mice infected intravenously with TIGR4 or Δ*idtr*.

Cytokine/Chemokine	TIGR4 (n = 5) (mean ± SEM^1^)	Δ*idtr* (n = 5) (mean ± SEM)	*P* value		
Eotaxin	8279.40±403.70	3691.57±224.38	<0.0001		
G-CSF	9850.12±0.0	235.22±8.83	<0.0001		
IFN-γ	14363.98±396.73	3.73±1.50	<0.0001		
IL-1β	201.93±11.09	17.67±4.14	<0.0001		
IL-6	16585.77±361.72	4.25±0.65	<0.0001		
IL-10	206.43±5.14	65.17±6.47	<0.0001		
IL-17	12.90±0.42	2.15±0.34	<0.0001		
MIP-2	2039.22±64.19	36.16±0.0		<0.0001	
KC	33583.04±2178.31	180.06±11.59		<0.0001	
MIP-1α (CCL3)	755.39±15.66	14.47±3.44		<0.0001	
RANTES (CCL5)	840.20±16.76	39.05±2.42		<0.0001	
TNF-α	194.59±4.02	1.10±0.20		<0.001	
IL-12p70	449.96±17.79	8.80±0.99		<0.001	
MCP-1 (CCL2)	63856.85±1601.45	31.46±6.63		<0.001	

1 Standard error of the mean.

## Discussion

The form and quantity of iron in humans varies significantly at different anatomical locations and it is likely that bacterial pathogens sense these differences, among other signals, and regulate gene expression in response. The exact mechanisms of iron acquisition and regulation in the pneumococcus are still largely unknown. However, the ability of this pathogen to colonize the highly iron-restricted environment of the nasopharynx and also cause invasive diseases in relatively iron-rich sites suggests that iron may be an important environmental signal for gene regulation.

A signature-tagged mutagenesis study in type 3 pneumococcus suggested a role for *smrB* (iron-dependent regulator) in pneumococcal virulence [25]. Although the authors proposed the gene designation *smrB*, we suggest the more associative *idtr* nomenclature. This gene was conserved in various unrelated pneumococcal strains and capsule types (data not shown).

We did not detect any significant difference in growth between wild-type and mutant either in presence or absence of iron in vitro. Additionally, no differences were observed between the mutant and wild-type in their ability to utilize a variety of iron sources (data not shown). The mutant forms clusters and aggregates in both the presence and absence of iron. These observations suggest that *idtr* has no significant role during pneumococcal growth in vitro but in some way affects bacterial cell-cell adhesion or daughter cell separation during cell division. TIGR4 and Δ*idtr* did not differ significantly in growth rates in blood following bacteremia up to 48 hours after infection. In relatively iron-rich environments such as blood *idtr* is not critical for pneumococcal growth. This observation parallels that seen in vitro in which the mutant was able to replicate as well as wild-type in presence of high iron concentration.

The contribution of *idtr* to pneumococcal sepsis was evaluated using a mouse model and both intravenous and intranasal inoculation. The Δ*idtr* mutant was significantly attenuated in the sepsis model by both routes of infection as compared to the parent strain but the more striking difference was observed with the intransal route of infection. We postulate that *idtr* is essential specifically during transition from the nasopharyngeal mucosa to submucosal tissue and blood. The Δ*idtr* mutant could be isolated from the nasopharynx two days after inoculation but not after day five, so lack of *idtr* may result in an even earlier deficiency, that is, an inability to efficiently colonize the nasopharynx. In either case it is likely that gene regulation by *idtr* is critical at mucosal surfaces where the concentration of extracellular iron in any form is exceedingly low.

Because increased mortality in mice infected with TIGR4 strain was not the result of more rapid cell growth in vivo, we selected ten known and putative virulence genes which might potentially be directly or indirectly regulated by *idtr*. We had previously studied these same genes in TIGR4 and found that they are differentially regulated in different anatomic sites in mouse models [6]. The expression of the selected genes was not markedly different between wild-type and the mutant in vitro but pronounced differences were noted during growth in vivo. Gene expression in Δ*idtr* was increased compared with wild-type in nasopharyngeal colonization and pneumonia, and was effectively unchanged during bacteremia for all genes except hemolysin. These results suggest that *idtr* does play a role in modulation of pneumococcal virulence. Based on these results we hypothesize that *idtr* contributes to repression of certain pneumococcal virulence-associated genes at mucosal surfaces and is de-repressed during bacteremia, possibly as a function of iron availability. An iron-dependent transcriptional regulator has been previously associated with virulence in a type 3 strain in pneumonia and bacteremia models by signature-tagged mutagenesis [25]. This study extends these findings to nasopharyngeal colonization and suggests that iron may be an important signal with effects on genes involved with virulence.

Sepsis results from systemic infection and the resultant systemic inflammatory responses [26]. The innate immune responses are critical inducers of sepsis syndrome in response to bacterial products and cellular components. Cytokines play a central role in regulation of the innate immune response and, therefore, in the manifestation of sepsis [27]. An exaggerated pro-inflammatory response which is the hall mark of sepsis is associated with high mortality both in humans and animal models. To uncover possible reasons for the improved survival of mice infected with Δ*idtr* we evaluated the host cytokine response. The concentration of 14 cytokines known to play an important role in invasive pneumococcal disease was evaluated in plasma and was found to be significantly decreased in plasma samples obtained from mice infected with Δ*idtr* as compared to TIGR4 infected mice. Most of the cytokines (Eotaxin, G-CSF, IFN-γ, IL-1β, IL-17, MIP-2, KC, MIP-1α, RANTES, TNF-α, IL-12, MCP-1) that were tested are pro-inflammatory cytokines except for IL-10 which is anti- inflammatory and IL-6 which has both pro [28] and anti- inflammatory effects [29]. Recent evidence indicates that both pro and anti- inflammatory responses are simultaneously regulated even in early stages of sepsis [30]. Increased levels of all the cytokines tested are associated with a poor prognosis in sepsis patients or animal models of sepsis [30]–[35]. Combined high levels of IL-10 and IL-6 are associated with a very high risk of death in sepsis patients [36].

This difference was not related to a faster growth rate and higher bacterial burden with TIGR4, as both wild-type and mutant were at the same approximate density in the blood at the time of cytokine sampling. These results imply that *idtr* not only modulates the bacterial virulence but also modulates the host response to pneumococcal infection. The mechanisms by which this modulation occurs remain to be determined. It is likely that *idtr* controls genes which encode pneumococcal surface-exposed components or other factors which interact with the host immune system. To our knowledge, this is the first report indicating a role of iron dependent transcription regulator in host immune response to pneumococcal infections. The role of iron-regulated bacterial genes in modulation of host responses has been reported for other Gram-positive pathogens. In *Staphylococcus aureus* the inactivation of *fur* is reported to be associated with increased nitric oxide sensitivity [37]. In *Mycobacterium smegmatis*, insertional inactivation of *ideR* (a homolog of *dtxR* and *idtr*) was shown to decrease production of manganese superoxide dismutase and catalase/peroxidase (katG), and increase susceptibility to killing by H_2_O_2_ [38].

IDTR has an important role in virulence and gene expression and its function is likely related to the form and quantity of available iron at different anatomic sites of the host. Invasive disease in humans follows translocation of pneumococci from mucosal surfaces of the nasopharynx to the lower respiratory tract and, in some cases, dissemination via blood. Environmental conditions are markedly different at each location and the concentrations of certain nutrients necessary for pneumococcal growth almost certainly function, by various pathways, to regulate bacterial gene expression. Future work will define the role of IDTR on global protein expression both in vitro and within a host and undoubtedly expand our understanding the complete subset of genes which are controlled either directly or indirectly by IDTR. Many of these gene products interact with host immune cells and contribute to pro-inflammatory cytokine responses and subsequent mortality in murine models. The identification of these bacterial gene products, and their specific interactions with the host immune system, will allow greater understanding of the pathogenesis of invasive pneumococcal infections and identify potential points at which intervention may be possible to reduce morbidity and mortality.

## Materials and Methods

### Bacterial strains and media

*S. pneumoniae* TIGR4, a capsular type 4 strain and an isogenic mutant deleted at *idtr* (described below) were used in all experiments. Bacteria from stocks stored at −80°C were used to inoculate chemically-defined medium (CDM) (JRH Bioscience, Lenexa, KS) [39] supplemented with 0.1% choline, 0.25% sodium bicarbonate and 0.073% cysteine. Iron-depleted CDM was prepared by treatment with 3% w/v Chelex-100® (Bio-Rad, Hercules, CA) for 20 h. Chelex-treated CDM was supplemented with MnSO_4_, MgSO_4_, and CaCl_2_ to a final concentration as that in CDM. All media were sterilized by filtration and stored at 4°C. Todd Hewitt yeast extract medium (THY) used in transformation of TIGR4 was made by adding 0.2% glucose, 0.2% CaCl_2_, and 0.02% bovine serum albumin (BSA) to THY medium and adjusted to pH 7.2–7.4 [40]. The trimethoprim resistance gene *tmp* was isolated from *E. coli* cells containing the *pko*T plasmid [41]. Trimethoprim (Tmp) was used at 50 µg/ml to select for transformants.

### *idtr* mutant construction

**Figure 5:**
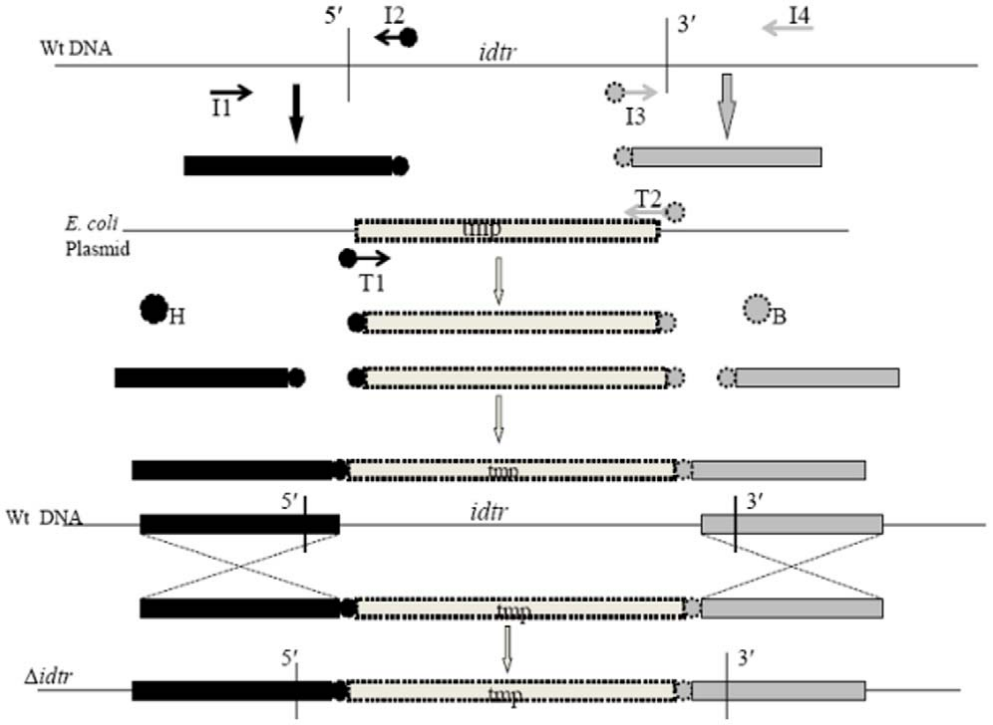
Schematic representation of Δ*idtr* construction. H-*HindIII*, B-*BamHI*. T1, T2 amplify the *tmp* cassette (495 bp); T1 and T2 have H and B at 5′ end. I1, I2 and I3, I4 amplify 5′ and 3′ end of *idtr*. I2 and I3 have H and B at 5′ end. I1, I2 amplify a 945 bp product and I3, I4 amplify a product of 489 bp.

An *idtr* mutant was constructed using PCR ligation mutagenesis as described by [42]. A schematic representation of the mutant construction is outlined in Figure 5. Briefly, *tmp* was amplified from *pko*T plasmid DNA (primers T1 and T2) and the flanking regions of *idtr* were amplified from TIGR4 genomic DNA (primers I1 and I2, I3 and I4) described in table 2. The PCR amplified and purified I1-I2, I3-I4 and the *tmp^r^* cassette were subjected to single and double digestion by *HindiIII* and *BamHI* respectively according to the manufacturer's protocol (Promega, Madison, WI), The digested PCR products were ligated using T4 DNA ligase (Promega, Madison, WI). The resulting construct (∼2 kb) was amplified using primers I1-I4 and was used to transform TIGR4 as previously described [25]. The double recombination event was selected by plating on plates containing 50 μg/ml of Tmp. Identification of Tmp-resistant mutants was confirmed by both PCR analysis and DNA sequencing.

**Table 2:** Primers used in mutagenesis.

Primers	Sequence (5′–3′)
I1	TCAATCGTTACCACTTTTCAACCAGTC
I2	NNAAGCTTAGATTTTCACTTTTCATTCGTT
I3	NNGGATCCAGTTTTGACATTCTCCATTATCT
I4	CAACTCTTGCTGTTTCACTTTCA
T1	NNNNAAGCTTATGAACCCGGAATCGGT
T2	NNNNGGATCCTTAGCCGTTACGACGCG

### Animal models of pneumococcal infection

All animal studies were performed on either 10–12 wk old CBA/CaHN-Btk*^xid^*/J or C57BL/6 mice obtained from the Jackson Laboratory (Bar Harbor, ME) and bred in the VA animal facility. This study was carried out in strict accordance with the recommendations in the Guide for the Care and Use of Laboratory Animals of the National Institutes of Health. The protocol was approved by the Institutional Animal Care and Use Committee at the VA Medical Center (assurance number: A3101-1). For all in vivo studies pneumococcal strains were administered either intranasally (i.n.) or intravenously (i.v.) as noted. Following infection animals were observed every twelve hours for signs of piloerection, inability to eat or drink, or failure to withdraw from threatening stimuli. Animals were weighed daily and any animal which exhibited any aforementioned behavior, or lost more than 15% of pre-infection body weight, was euthanized. For intranasal infection, a suspension of mid-exponential phase TIGR4 or Δ*idtr* (10^6^ CFU) in PBS was delivered into the nares of anesthetized mice (20 µl per mouse) as previously described [43]. At this volume and cell number pneumococci remain localized to the nasopharynx. Intravenous inoculation was performed by injecting 10^5^ CFU of TIGR4 or Δ*idtr* in 100 µl of PBS into the tail vein. Inocula for each experiment were confirmed by serial dilution and plate counting.

### In vitro growth of TIGR4 and Δ*idtr*

TIGR4 or Δ*idtr* was inoculated into CDM and incubated at 37°C until cells reached mid-exponential phase growth (O.D_600_ of 0.4–0.6)_._ The cells were harvested by centrifugation, washed twice with sterile PBS and subcultured into CDM and iron-depleted CDM. Bacterial growth was monitored by measuring absorbance at 600 nm after brief vortexing and cell morphology was examined by Gram staining. Following all experiments terminal sub-cultures were performed by plating on to blood agar plates (BAP) and testing for α-hemolysis and optochin sensitivity to check the purity and identity of the cultures. Cultures of Δ*idtr* were terminally sub-cultured on plates containing 50 µg/ml Tmp.

### In vivo growth of TIGR4 and Δ*idtr*

Blood samples were collected by retro-orbital bleeding from mice inoculated intravenously with either TIGR4 or Δ*idtr* at 3, 6, 12, 24, 36 and 48 h after infection. Bacterial density was determined by plating serially diluted blood samples on BAP and incubating 18–24 hrs at 37°C in 5% CO_2_.

### In vitro and in vivo gene expression

The expression of ten characterized or putative pneumococcal genes associated with virulence was evaluated as described previously [6]. For in vitro experiments TIGR4 and Δ*idtr* were grown in CDM at 37°C until mid-exponential phase. Nasopharyngeal washes and blood samples were obtained as previously described [43] and lung homogenates were collected as described [44]. Blood samples were collected at 48 hours for isolation of total RNA from bacteria and sera was collected for cytokine analysis. Animals inoculated with PBS were used as negative controls. RNA was isolated and gene expression was measured by quantitative RT-PCR as previously described [6]. Relative gene expression was analyzed using PFAFFL method [45] and fold changes were normalized to 16S rRNA.

### Host cytokine response in sepsis

The concentrations of 14 cytokines and chemokines (eotaxin, G-CSF, IFN-γ, IL-1β, IL-6, IL-10, 1L-17, MIP-2, KC, MIP-1α, RANTES, TNF-α, IL-12p70, MCP-1) were analyzed in plasma samples obtained from two groups of 5 mice each infected intravenously with TIGR4 or Δ*idtr* as described above. The blood samples from which plasma was obtained were collected 48 hours after infection. Cytokine and chemokine concentrations were determined using Milliplex MAP Assay kits which are based on the Luminex xMAP technology (Millipore Corp., Billierica, MA) using standards and controls for each cytokine and chemokine provided by the manufacturer. All samples were evaluated in duplicates at two different dilutions.

### Statistical analysis

Survival was plotted with Kaplan-Meier curves and differences were compared using a log rank test. Cytokine and chemokine levels were compared using an unpaired *t* test (Graph Pad Prism 4.0, La Jolla, CA). A P value of <0.05 was considered significant.
