# Correction: Role of an Iron-Dependent Transcriptional Regulator in the Pathogenesis and Host Response to Infection with *Streptococcus pneumoniae*

**DOI:** 10.1371/annotation/0b2b0a8b-fb01-410a-8416-f961e92c9fac

**Published:** 2013-10-01

**Authors:** Radha Gupta, Minny Bhatty, Edwin Swiatlo, Bindu Nanduri

The first two authors, Radha Gupta and Minny Bhatty, should be noted as contributing equally to this work and sharing in first authorship. 

